# Epidemiologic Study of *Vibrio vulnificus* Infections by Using Variable Number Tandem Repeats

**DOI:** 10.3201/eid1508.080839

**Published:** 2009-08

**Authors:** Yoav Y. Broza, Yael Danin-Poleg, Larisa Lerner, Lea Valinsky, Meir Broza, Yechezkel Kashi

**Affiliations:** Technion-Israel Institute of Technology, Haifa, Israel (Y.Y. Broza, Y. Danin-Poleg, Y. Kashi); Ministry of Health, Jerusalem, Israel (L. Lerner, L. Valinsky); University of Haifa, Oranim, Tivon, Israel (M. Broza)

**Keywords:** Vibrio vulnificus, bacteria, epidemiology, variable number tandem repeats, VNTR, bacterial typing, microsatellites, single-sequence repeats, SSR, dispatch

## Abstract

A 3-year environmental and clinical *Vibrio vulnificus* survey using simple-sequence repeats typing shows that *V. vulnificus* biotype 3 constitutes ≈21% of the bacterium population in tested aquaculture ponds as opposed to ≈86% of clinical cases. Simple-sequence repeats proved to be a useful epidemiologic tool, providing information on the environmental source of the pathogen.

*Vibrio vulnificus* is a highly invasive human pathogen and presents a food safety issue worldwide. Human infections caused by *V. vulnificus* are primarily caused by contaminated seafood consumption or contaminated skin wounds, which can lead to septicemia, wound infections, and high hospitalization and fatality rates (*1*,*2*). *V. vulnificus* strains are biochemically divided into 3 biotypes (BTs). BT3, found in Israel, is associated with infections caused by contaminated fish (*3*). Until now, only 3 BT3 isolates had been isolated from the environment in direct contrast with their large clinical numbers (*3*,*4*).

BT3 is a clonal group, which various molecular methods have failed to differentiate among its strains, (*4*,*5*) with the exceptions of rep-PCR (*6*), simple-sequence repeats (SSR) (*7*), and recently pulsed-field gel electrophoresis (PFGE) (*8*). SSR analysis of *V. vulnificus* was highly discriminative among BT3 strains (*7*). These SSR markers have been used for typing and for epidemiologic studies in many bacterial species (*9*,*10*). We present results from a 3-year monitoring program of clinical and environmental *V. vulnificus* using SSR as an epidemiologic genotyping tool.

## The Study

A total of 414 *V. vulnificus* isolates were studied, including a reference panel of 32 strains previously studied (*7*). A total of 360 environmental *V. vulnificus* isolates were successfully retrieved from September 2004 through October 2006 from artificial fish ponds and stores in the western Galilee region of Israel (from 21 samplings), and 22 clinical isolates were retrieved from nearby hospitals during matching years (Tables 1, 2). Fish samples were collected and gills and fins/scales were pooled from ≈10 *Tilapia* spp. (300–400 g). Each sample was incubated in 0.5 L modified alkaline peptone water with 4% NaCl, pH 6.9, at 37^o^C for 16 to 18 h. Samples were diluted in saline and streaked on thiosulfate-citrate-bile-salts-sucrose (TCBS) agar. Suspected colonies were further grown on chromogenic agar (CHROMagar Microbiology, Paris, France), and validated by amplification of *V. vulnificus*–specific gene vvh (*7*). All *V. vulnificus* colonies were green on TCBS agar. Notably, not all bacterial isolates showed the expected turquoise-colonies on CHROMagar but rather pale white colonies. The latter colonies were further identified as BT3. All other isolates, which were BT1, showed the expected turquoise-colony phenotype. No BT2 isolates were found. However, 6 previously studied isolates (*7*) showed the expected turquoise-colony.

**Table 1:** *Vibrio vulnificus* isolates from Israel that were genetically analyzed, 2003–2006*

Isolate identification	Biotype	Date	Origin/hospital name	IMH no.†
Environmental				
VVyb1	3	2004	Store: yb1-yb53 Pond: yb54-yb58	
VVyb2–VVyb58, (VVyb38, VVyb45 missing)	1			
VVyb63, VVyb66, VVyb67 VVyb71–VVyb73, VVyb83, VVyb86–Vvyb93, VVyb95–Vvyb109, VVyb111–VVyb126	3	2005	Store: yb87-yb126, yb158-yb193 Pond: yb62-yb86	
VVyb59–VVyb62, VVyb64,VVyb65, VVyb68–VVyb70, VVyb74–VVyb82, VVyb84, VVyb85, VVyb159, VVyb162–VVyb164, VVyb167–VVyb170, VVyb172–VVyb182, VVyb187, VVyb189–VVyb193	1			
VVyb94, VVyb110, VVyb158, VVyb160, VVyb161, VVyb165, VVyb166, VVyb171, VVyb183–VVyb186, VVyb188	ND			
VVyb127–Vyb133, VVyb137–VVyb157	3	2006	Store: yb127-yb134, yb194-yb206 Pond: yb135-yb157, yb207-yb221	
VVyb134–VVyb136, VVyb195–VVyb208, VVyb210–VVyb216, VVyb218–VVyb221, (VVyb201, VVyb212 missing)	1			
VVyb209, VVyb217	ND			
v232‡	3	2003 Dec	Fish	8/03e
Clinical§				
v233	3	2006 May	Rivka Ziv	1/06
v234	3	2006 Sep	HaEmek	2/06
v235	ND	2006 Oct	Western Galilee	3/06
v236	3	2005 Feb	HaEmek	1/05
v237	3	2005 Jun	Western Galilee	2/05
v238	3	2005 Jun	Western Galilee	5/05
v239	3	2005 Aug	Rambam	6/05
v240	3	2005 Oct	Western Galilee	7/05
v241	3	2005 Nov	Rambam	8/05
v242	3	2005 Nov	Rambam	9/05
v243	3	2005 Dec	Carmel	10/05
v244	3	2005 Nov	Western Galilee	11/05
v245	3	2005 Jun	Carmel	3/05
v246	ND†	2005 Jun	Rambam	4/05
v247	3	2004 Jun	HaEmek	2/04
v248	3	2004 Jun	HaEmek	3/04
v249	3	2004 Jun	HaEmek	4/04
v250	3	2004 Jul	Rambam	5/04
v251	3	2004 Aug	Western Galilee	6/04
v252	1	2004 Aug	Western Galilee	7/04
v253	3	2004 Oct	Carmel	9/04
v254	3	2003 Dec	Barzilai	8/03

*IMH, Israeli Ministry of Health; ND, not determined. †All clinical isolates are part of the IMH collection. ‡Previously studied (*7*). §All clinical isolates are associated with fish.

**Table 2:** Environmental *Vibrio vulnificus* isolates obtained in Israel from artificial fish ponds and fish stores, 2004–2006

Dates	No. samples	No. isolates	No. V. vulnificus*	Biotype 3, %	Biotype 1, %†
2004 Sep–Oct	7	58	58	2	98
2005 May–Oct	5	280	166	28	72
2006 Mar–Oct	9	251	136	21	79
Total	21	589	360		

*Tested by specific amplification of *vvh* gene. †Including isolates not determined.

**Figure 1:**
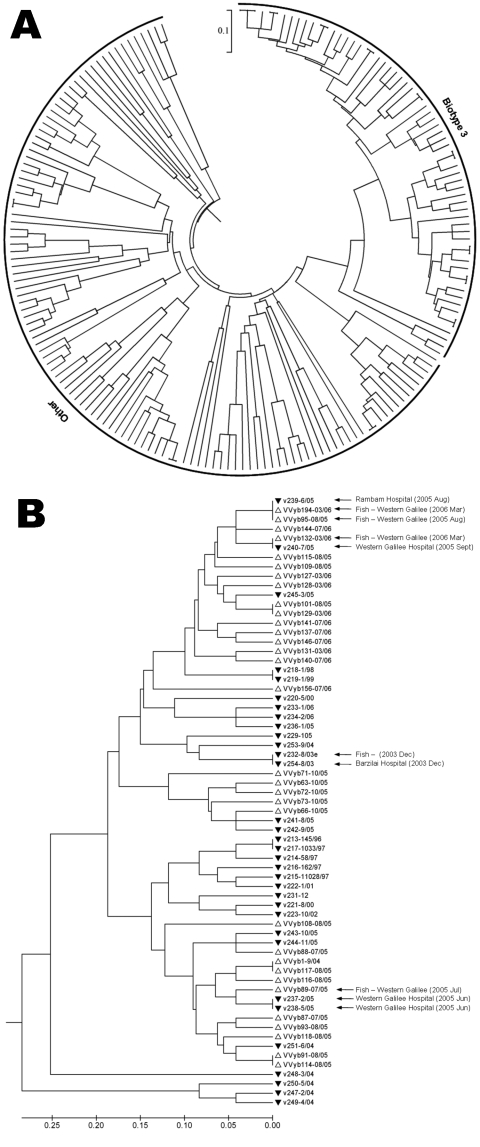
A) Genetic relationships based on simple-sequence repeat (SSR) variation data among 183 *Vibrio vulnificus* isolates including 135 new environmental, 22 new clinical, and 26 previously studied isolates. B) A subtree enlargement of panel A displaying a set of 65 *V. vulnificus* biotype 3 isolates. Similar clinical and environmental isolates, showing an epidemiologic connection, are indicated by arrows. The genetic-distance matrix was generated based on 212 polymorphic points (the sum of alleles across 12 SSR loci). Genetic relationships are based on unweighted pair group method with arithmetic mean cluster analysis of SSR variation using MEGA4 software (*11*). Scale bar represents genetic distance.

SSRs were used to genetically characterize 254 clinical and environmental *V. vulnificus* isolates (Table 1), including 32 previously studied isolates (*7*). DNA extraction, PCR and primers, SSR sizing, and statistical analysis were conducted as previously described (*7*). Capillary electrophoresis was performed by using a 3130 Genetic Analyzer and analyzed with GeneMapper-v4.0 (Applied-Biosystems Inc., Foster City, CA, USA). Two to 34 alleles were detected at the 12 SSR loci among the isolates. Environmental isolates were selected from 21 samples with an average 8.5 isolates per enrichment. We removed 71 isolates that had identical SSR genotypes and originated from the same enrichment from the analysis because they were probably clones. Thus, 183 isolates were discriminated to 170 SSR types. SSR variation data was used to calculate genetic relationships among isolates. A genetic distance matrix was generated followed by cluster analysis (*7*). The resulting dendrogram (Figure 1) showed clear separation between BT3 isolates and the others (average genetic distance of 0.825 ± 0.101). Genetic distances among BT3 isolates were rather low (average 0.369 ± 0.174) relative to high genetic distances (average 0.804 ± 0.149) found among isolates of the other biotypes, in accordance with our previous analysis of 32 isolates (*7*). The new studied isolates showed a variety of SSR genotypes and were spread throughout the dendrogram (Figure 1). Further analysis using eBURST (*12*) showed similar grouping results (data not shown) (*7*).

Differentiation of SSR alleles results at locus VV0401 into environmental types (E-types, >12 repeats), and clinical types (C-types, <10 repeats) was tested (*13*). Of the clinical isolates, 44 isolates (98%) exhibited the expected C-type allele/repeat but only 69 isolates (33%) of the environmental isolates showed the E-type allele/repeat, rejecting the null hypothesis (p<0.0001), using Pearson χ^2^ test. Notably, 97% of 110 BT3 isolates (clinical/environmental) showed the C-type allele/repeat, in contrast to BT1 isolates (72 isolates, 2%). If, C-type allele/repeat at VV0401 is an indication of potential pathogenicity of *V. vulnificus* strains, then our results further support the high virulence of BT3. However, additional studies are needed to confirm the relationship of this locus to pathogenicity.

Three clinical BT3 isolates exhibited identical SSR genotypes and 2 clinical BT3 isolates had a genotype related to 5 environmental isolates sampled on related dates from nearby regional areas (Figure 1, panel B). One clinical BT3 isolate v239 (August 2005) showed SSR genotypes identical to the environmental isolate VVyb95 obtained in the same month (August 2005) and to VVyb194 (obtained in March 2006). A second BT3 clinical isolate v240 (October 2005) and the fish-pond isolate VVyb132 (March 2006) had identical SSR genotypes. Moreover, these 2 results suggest survival of *V. vulnificus* strains through the winter season, either in a viable nonculturable state (*14*) or as viable cells in sediment that can serve as a shelter for some subpopulations (*15*). The latter scenario is more probable in artificial fish ponds because water circulation is high throughout the growth period and pond sediment remains untouched. A third clinical isolate was analyzed earlier and showed an epidemiologic connection: v254 isolate was obtained from an injured woman (injured by a fish) in December 2003. Analysis of microbial flora on the fish (found in the woman’s freezer) identified a *V. vulnificus* BT3 isolate, v232. These 2 isolates showed identical SSR genotypes, confirming the fish as the origin of infection (Figure 1, panel B). Additionally, clinical BT1 isolate v252 (August 2004), showed similar SSR genotypes, 1 repeat difference in 1 locus, to environmental isolate VVyb50 (October 2004).

**Figure 2:**
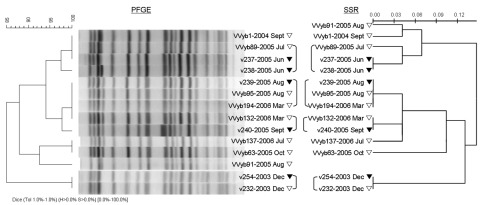
Genetic relationships showing the epidemiologic connection among 12 clinical and environmental *Vibrio vulnificus* biotype 3 isolates based on pulsed-field gel electrophoresis (PFGE) analysis compared to analysis at 12 single-sequence repeat (SSR) loci. PFGE profiles were compared by using the Dice coefficient followed by unweighted pair group method with arithmetic mean clustering (tolerance, 1.0%). Scale bars represent pattern similarity (% for PFGE and genetic distance for SSR).

To strengthen our typing results, we compared the epidemiologic SSR results to those of PFGE in 12 representative BT3 isolates. PFGE was performed and analyzed as described previously (*8*). Results for PFGE were generally similar to results for SSR (Figure 2). PFGE patterns were similar (>85%) between isolates. Identical PFGE patterns and SSR genotypes were seen in isolates v239 (clinical) and VVyb95, VVyb194 (environmental), as were isolates v232 (fish) and v254 (clinical). Identical PFGE patterns and single-locus variants in SSR genotypes were seen in clinical isolate v237 and the environmental isolates VVyb89 and VVyb1. Notably, VVyb1, which was isolated a year earlier, differentiated from VVyb89 by another single repeat in an additional single locus, confirming higher resolution of SSR method. Finally, identical SSR genotypes and PFGE patterns that differed by 1 band were seen in v240 and VVyb132.

## Conclusions

The developed isolation and enrichment procedures obtained large numbers of BT3 and BT1 from the environment. Results showed that although BT3 makes up only ≈21% of the *V. vulnificus* isolates from fish, BT3 accounts for ≈86% of the clinical cases and thus could imply high pathogenicity for this group (*4*). Genetic analysis of this large survey confirms the distinctness (clonality) (*5*) of BT3 and the high resolution power of the SSR (*7*).

SSR genotyping of *V. vulnificus* was used to determine the genetic relatedness between clinical and environmental isolates and identify the source of contamination. SSR can serve as an epidemiologic tool to indicate the infection source of pathogens such as *V. vulnificus*, and can potentially provide knowledge for preventive steps in terms of public health.
